# Supplementary material for: A Carrier Female Manifesting an Unusual X-Linked Retinoschisis Phenotype Associated with the Pathogenic Variant c.266delA, p.(Tyr89LeufsTer37) in RS1, and Skewed X-Inactivation
Source: Genes (Basel). 2023 May 29;14(6):1193. doi: 10.3390/genes14061193 (PMC10298380; doi:10.3390/genes14061193)
Supplement: Supplementary file 1 [file genes-14-01193-s001.zip › genes-2382826-supplementary.pdf]

Table S1. Information on target genes and NGS coverage data for proband.

Gene	Reference sequence/s	% ROI Covered by NGS (Depth >30x)
ABCA4	NM_000350.2	99.8
ABHD12	NM_015600.4<br>NM_001042472.2	100
ADAM9	NM_003816.2	95
AIPL1	NM_014336.3	99.5
ARL6	NM_032146.3	78.7
BBS1	NM_024649.4	100
BEST1	NM_004183.3<br>NM_001139443.1	99.1
C1QTNF5	NM_015645.3	96.9
C2ORF71	NM_001029883.1	97.9
C8ORF37	NM_177965.3	94.9
CA4	NM_000717.3	96
CABP4	NM_145200.3	100
CACNA1F	NM_005183.2<br>NM_001256790.2	97.5
CACNA2D4	NM_172364.4	98.2
CAPN5	NM_004055.4	99.7
CC2D2A	NM_001080522.2<br>NM_020785.2<br>NM_001164720.1	96.6
CDHR1	NM_033100.2<br>NM_001171971.1	99.9
CEP290	NM_025114.3	90.5
CERKL	NM_001030311.2	93.7
CHM	NM_000390.2<br>NM_001145414.1	95.5
CLRN1	NM_001195794.1<br>NM_052995.2<br>NM_001256819.1	99.7
CNGA1	NM_001142564.1	97.8
CNGA3	NM_001298.2	100
CNGB1	NM_001297.4	99.8
CNGB3	NM_019098.4	93.8
CRB1	NM_201253.2	98.4
CRX	NM_000554.4	100
CYP4V2	NM_207352.3	99.6
DHDDS	NM_024887.3	99.6
EYS	NM_001142800.1<br>NM_198283.1<br>NM_001142801.1	97.3
FAM161A	NM_001201543.1	95.9
FLVCR1	NM_014053.2	99.8
FSCN2	NM_001077182.2	89.3
FZD4	NM_012193.3	95.4
GNAT1	NM_144499.2	100
GNPTG	NM_032520.4	96.2
GPR179	NM_001004334.2	99.9
GRK1	NM_002929.2	98.9
GRM6	NM_000843.3	93.5
GUCA1A	NM_000409.3	100
GUCA1B	NM_002098.5	100
GUCY2D	NM_000180.3	97.5
IDH3B	NM_006899.3<br>NM_001258384.1<br>NM_174855.2	100
IMPDH1	NM_000883.3<br>NM_001102605.1	100
IMPG2	NM_016247.3	99.6
IQCB1	NM_001023570.2	94.5
KCNJ13	NM_002242.4<br>NM_001172416.1	99.2
KCNV2	NM_133497.3	100
KLHL7	NM_001031710.2<br>NM_001172428.1	100
LCA5	NM_001122769.2	94.1
LRAT	NM_004744.3	100
LRIT3	NM_198506.4	99.1
LRP5	NM_002335.3	97.2
MAK	NM_001242957.1	92.4
MERTK	NM_006343.2	98.1
NDP	NM_000266.3	100
NMNAT1	NM_022787.3	97.5
NR2E3	NM_014249.2	99.1
NRL	NM_006177.3	97.9
NYX	NM_022567.2	64
OFD1	NM_003611.2	99.4
OTX2	NM_021728.3	100
PDE6A	NM_000440.2	99.4
PDE6B	NM_000283.3	98.6
PDE6C	NM_006204.3	94.5
PDE6G	NM_002602.3	100
PDE6H	NM_006205.2	100
PITPNM3	NM_031220.3	97.7
POC1B	NM_172240.2	99.1
PRCD	NM_001077620.2	93.3
PROM1	NM_006017.2	97.2
PRPF3	NM_004698.2	97
PRPF31	NM_015629.3	100
PRPF6	NM_012469.3	100
PRPF8	NM_006445.4	99.8
PRPH2	NM_000322.4	100
RAB28	NM_001017979.2<br>NM_004249.3,<br>NM_001159601.1	100
RAX2	NM_032753.3	88.4
RBP3	NM_002900.2	99.8
RBP4	NM_006744.3	100
RD3	NM_183059.2	100
RDH12	NM_152443.2	100
RDH5	NM_001199771.1	98.9
RGR	NM_002921.3	100
RHO	NM_000539.3	100
RIMS1	NM_014989.5<br>NM_001168407.1<br>NM_001168410.1	98.5
RLBP1	NM_000326.4	99.3
ROM1	NM_000327.3	94.6
RP1	NM_006269.1	99.2
RP2	NM_006915.2	92.7
RP9	NM_203288.1	80.7
RPE65	NM_000329.2	98.7
RPGR	NM_001034853.1<br>NM_000328.2	95.4
RPGRIP1	NM_020366.3	99.5
RPGRIP1L	NM_015272.2	97.7
RS1	NM_000330.3	100
SAG	NM_000541.4	94
SEMA4A	NM_022367.3	100
SLC24A1	NM_004727.2<br>NM_001254740.1	98
SNRNP200	NM_014014.4	99.1
SPATA7	NM_018418.4<br>NM_001040428.3	95.9
TOPORS	NM_005802.4	100
TRPM1	NM_001252020.1<br>NM_001252024.1<br>NM_001252030.1<br>NM_002420.5	98.9
TSPAN12	NM_012338.3	98.9
TTC8	NM_144596.3	97.3
TTPA	NM_000370.3	89.2
TULP1	NM_003322.3	96.8
UNC119	NM_005148.3<br>NM_054035.2	99.1
USH2A	NM_206933.2<br>NM_007123.5	99.2
WDR19	NM_025132.3	97.9
ZNF513	NM_015069.2	95.9
